# Long-Term Health Related Quality of Life following Intensive Care during Treatment for Haematological Malignancies

**DOI:** 10.1371/journal.pone.0087779

**Published:** 2014-01-31

**Authors:** Maarten van Vliet, Mark van den Boogaard, J. Peter Donnelly, Andrea W. M. Evers, Nicole M. A. Blijlevens, Peter Pickkers

**Affiliations:** 1 Department of Haematology, Radboud University Medical Center, Nijmegen, The Netherlands; 2 Department of Intensive Care Medicine, Radboud University Medical Center, Nijmegen, The Netherlands; 3 Department of Medical Psychology, Radboud University Medical Center, Nijmegen, The Netherlands; 4 Institute of Psychology; Health, Medical and Neuropsychology Unit, Leiden University, Leiden, The Netherlands; The James Cook University Hospital, United Kingdom

## Abstract

**Objective:**

Long-term health-related quality of life (HRQoL) was determined for patients admitted to the haematology ward who needed intensive care treatment (H-IC+) and compared with those who did not (H-IC−) as well as with that for patients admitted to the general ICU (nH-IC+).

**Methods:**

A cross-sectional study was carried out median 18 months after admission by employing the short form-36, checklist for individual strength, cognitive failure questionnaire and hospital anxiety and depression scale.

**Results:**

27 (79%) of the 34 H-IC+ patients approached, and 93 (85%) of the 109 H-IC− patients approached replied. Data were adjusted for relevant covariates and matched with those of 149 patients in the general ICU. Apart from the lower role-physical functioning score for H-IC+ (*P* = 0.04) no other differences were found between H-IC+ and H-IC−. Groups H-IC+ and nH-IC+ evaluated their HRQoL on SF-36 similarly, except for the lower aggregated physical component summary (PCS) for H-IC+ (*P*<0.0001). After adjusting for PCS, no significant differences in CIS, CFQ and HADS were observed between the groups.

**Conclusions:**

Eighteen months after admission, patients treated for haematological malignancies reported similar HRQoL, whether or not they had received intensive care treatment, but reported a lower PCS than those of patients in the general ICU. Hence, there is no reason to assume that admission to the ICU has a negative impact on long-term HRQoL, so this should not affect the decision whether or not to transfer patients with haematological malignancies to the ICU.

## Introduction

Survivors of critical illnesses are frequently left with a legacy of long-term physical, neuropsychiatric and quality of life impairments [1]. Patients who survive critical illness report significantly lower health-related quality of life (HRQoL) after a year compared to their well being before intensive care unit (ICU) admission as well as that of the general population [2], [3].

Treatment of patients with haematological malignancies has become increasingly intensive consisting of several cycles of high-dose chemotherapy, often followed by an allogeneic or autologous hematopoietic stem cell transplant (HSCT). As a consequence, these patients are at risk for critical illness. In the last decade the contribution of ICU's during treatment of haematological malignancies has significantly improved [4]. ICU physicians have been successful in improving survival beyond the acute stage of critical illness by emphasizing the importance of early recognition of clinical deterioration. Improved insight into the treatment of sepsis and the introduction of non-invasive and protective positive pressure ventilation strategies to overcome respiratory failure have also played a role [5]–[7].

Long-term HRQoL of patients being treated for haematological malignancies is relevant to assist physicians in their decision whether or not to admit the patient to the ICU. Recent reports are conflicting, as both persistent impaired HRQoL [3] and restorement of HRQoL 90 days following ICU discharge in 80% of survivors was determined [8]. No comparisons to haematological patients that did not need intensive care and non-haematological ICU patients were made.

With the current study we aim to determine long-term self-reported HRQoL, including fatigue, cognitive functioning, anxiety and depression of patients being treated for a haematological disease who were given intensive care and to compare these HRQoL scores with those of haematological patients who did not receive intensive care as well as those of a group of general medical ICU patients.

## Patients and Methods

### Ethics statement

The Committee on Research Involving Human Subjects (Arnhem-Nijmegen region CMO) approved the study protocol (study number 2010/306) and waived the need for informed consent since the objective of this study was to evaluate regular patient care. Patient privacy was guaranteed as all data were anonimised and evaluated in a blinded fashion.

### Patients with haematological disease

**Figure 1:**
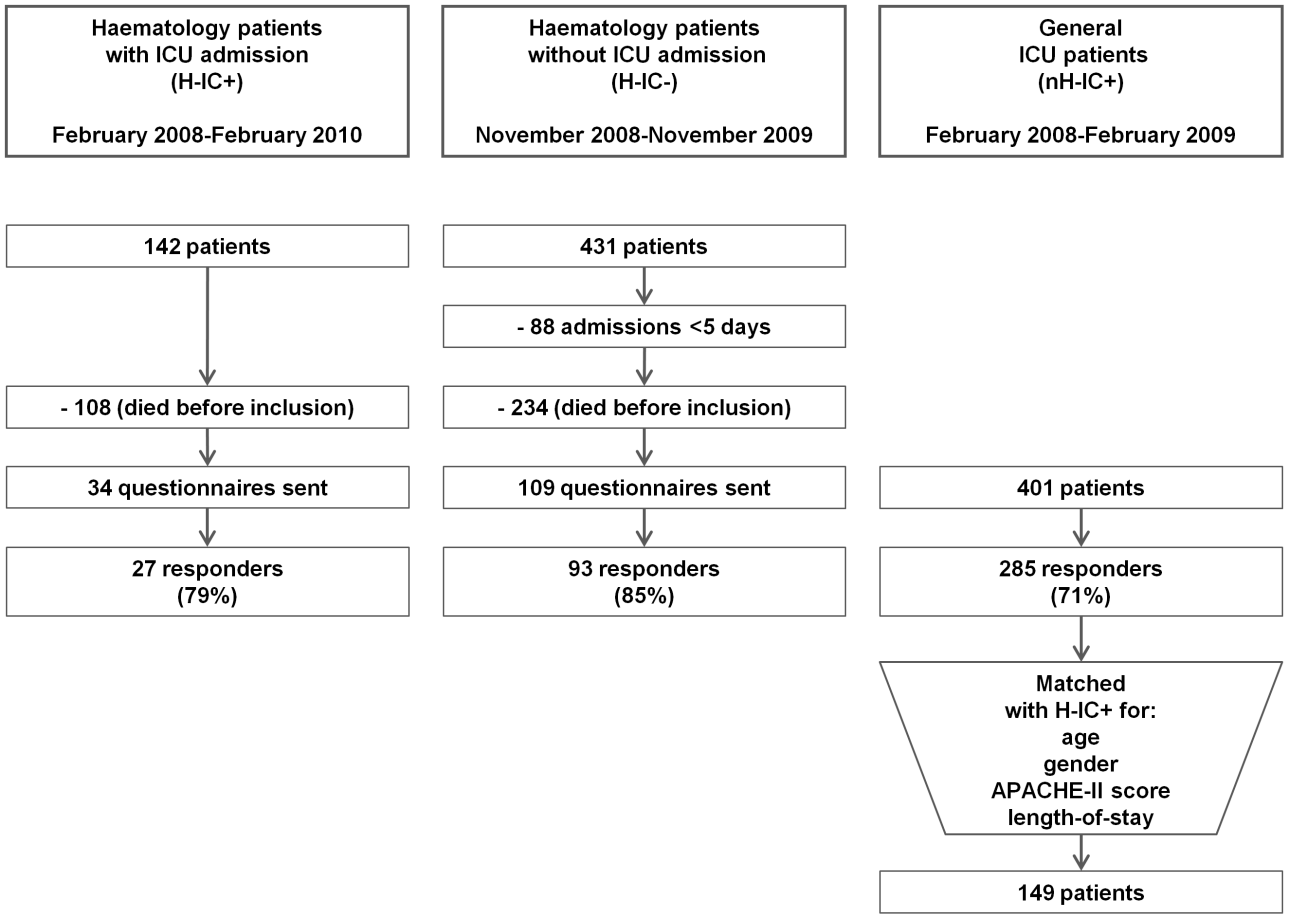
Flowchart on inclusion. Enclosed as file ‘figure 1’.

All consecutive patients who were admitted for five days or more to our tertiary care medical centre for treatment of a haematological malignancy during a 1 year period were eligible for the survey. Survivors were divided into two groups depending upon whether or not they had been admitted to the ICU. A HRQoL questionnaire was sent to them a median of 18 months after admission (range of 12–24 months). This same procedure was carried out twice on two consecutive years for those who were admitted to the ICU during treatment in order to enlarge the group (Figure 1). Non-responders were sent a reminder six weeks later. Demographic and medical data were collected from the patient files.

### General ICU patients

Data of general ICU patients without a haematological malignancy were retrieved from a group of 401 medical admission patients between February 2008 and February 2009. These patients were approached in a similar way as the haematological groups and non-responders were sent a reminder six weeks later. A total of 285 (71%) patients responded and 149 of these were meticulously matched with their haematological ICU counterparts for age, gender, APACHE-II score and ICU length of stay. The methods of HRQoL measurement for this group was similar to that for the haematological groups [9].

### ICU characteristics

There was no explicit ICU admission policy at the time and the decision to admit a patient to one of the level 3 general ICUs was made by the senior haematologists and intensivists. ICU characteristics and reason for ICU admission were extracted from patient files.

### Questionnaires

The following four different validated questionnaires were used to measure health related quality of life. The short form-36 (SF-36) contains 8 multi-item dimensions covering various aspects of physical, social, emotional and mental health. Missing values were imputed according to the health survey manual [10] and aggregated summary scores were expressed in a physical component summary (PCS) and a mental component summary (MCS) ranging between 0–100, a higher score indicating a higher level of functioning [11]. The shortlist of the checklist individual strength-fatigue (CIS-fatigue), consisting of 8 questions scoring on a 7-point Likert scale. The range of CIS-fatigue is 8–56, a higher score indicating more pronounced fatigue [12]. The validated Dutch translation of the cognitive failure questionnaire (CFQ) is self-reported [13] and consists of 25 questions on memory, distractibility, social blunders and names [14]. Each question was scored on a 5-point Likert scale. The total score on the CFQ ranges from 0–100, a higher score indicating more self-reported cognitive impairment. The hospital anxiety and depression scale (HADS) is a 14-item self-reporting measure of psychological distress and widely used for cancer patients [15]. The HADS has two subscales (anxiety and depression), each ranging from 0 to 21. Each item is rated on a scale from 0 (‘not at all’) to 3 (‘very much’) and higher scores indicate more anxiety and depression [16]. The non-haematological ICU patients did not receive the HADS questionnaire.

Thus, our self-reported HRQoL survey consisted of a total of 83 questions. The data obtained were recoded and subsequently scored according to their manuals. To guarantee patients' privacy, the survey was sent out anonymously and assigned a number. This allowed the primary and supervising investigator to match the returned survey with the patient's registry number held in a separate confidential database.

### Statistical analysis

All data were analyzed using SPSS version 20.0.0.1 (SPSS, Chicago, IL). The t-test was used for normally distributed variables, Mann-Whitney U test for variables that were not normally distributed and the Chi-square test for binary variables. Correlations between physical component summary and mental component summary were determined using Pearson's correlation coefficient.

Significant differences between the variables were considered as covariates. Since data were not normally distributed, a log-transformation was performed in order to perform a multivariate analysis of covariance. Since this was an exploratory study, and to increase sensitivity for putative differences between the groups, there was no correction for multiple testing. Statistical significance was defined as a *P*-value <0.05.

## Results

### Characteristics of patients with haematological disease

In total 573 patients were eligible, of which 143 patients could be approached for the study (Figure 1). Mortality during this long-term follow-up was significantly higher for H-IC+ (108/142, 76%), compared to group H-IC− (234/431, 45%) (*P*<0.0001).

In H-IC+ 34 questionnaires were sent out and 27 questionnaires (79%) were completed, median 15 months [Inter Quartile Range (IQR) 12–20] following admission. The mean APACHE-II score was 18.5±9.2 (Table 1). In total 109 questionnaires were sent to haematological patients that did not need ICU treatment and 93 questionnaires (85%) were completed, median 16 months [IQR 13–20] following admission. In both groups the proportion of patients admitted for an allogeneic or autologous hematopoietic stem cell transplant, chemotherapy or complications were comparable. No significant differences in demographic characteristics were found between the groups, except for the proportion of acute leukaemia/MDS which was higher in H-IC+ (*P* = 0.008, Table 1). Hospital length of stay was significantly longer for H-IC+ (median 33 days [interquartile range 25–42]), compared to H-IC− (21 days [11]–[27], *P*<0.001) or nH-IC+ (18 days [10–37], *P*<0.001).

**Table 1:** Demographic characteristics of responding patients with and without ICU admission.

	Haematology patients with ICU admission (N = 27)	Haematology patients without ICU admission (N = 93)	General ICU patients (N = 149)
Age	52.8±14.2	53.5±13.3	56.9±16.7
Gender (M)	17 (63%)	54 (58%)	72 (48%)
APACHE-II score	18.5±9.2	n.a.	19.0±5.4
LOS-Hospital (days)	33 [25–42]^a^	21 [11–27]	18 [10–37]^b^
LOS-ICU (days)	5 [2–10]	n.a.	4 [2–10]
Diagnosis (N,%):			
Acute leukaemia and MDS (AML, ALL and MDS)	13 (48)^a^	20 (22)	
Malignant lymphoma (NHL, M. Hodgkin and M. Waldenström)	7 (26)	28 (30)	n.a.
Multiple myeloma	4 (15)	28 (30)	
Chronic leukaemia (CML and CLL)	1 (4)	13 (14)	
Other	2 (7)	4 (4)	
Hospital admission reason (N,%):			
Allogeneic HSCT (SIB, VUD and SIB-RIC)	6 (22)	15 (16)	
Autologous HSCT	4 (15)	29 (31)	n.a
Non-HSCT chemotherapy	7 (26)	16 (17)	
Complications (after non-HSCT chemotherapy, after HSCT or without preceding treatment)	10 (37)	33 (36)	

Data are expressed as mean with (±) standard deviation or median with IQR unless other reported.

a statistically significantly different (*P*<0.05) compared with haematology patients without ICU admission.

b statistically significantly different (*P*<0.05) compared with haematology patients with ICU admission.

### Characteristics of general ICU patients

In total 149 general ICU patients were matched with the haematological patients that needed ICU treatment. Twenty-six (17%) patients had a proven infection of which 18 patients had a sepsis. The mean APACHE-II score was 19±5.4 (Table 1).

### Health Related Quality of Life Questionnaires

Haematological diagnosis and length of hospital stay served as covariates for the SF-36 analysis and were complemented by the Physical Component Summary as covariate for the analyses of CIS-fatigue, CFQ and HADS differences between the groups.

### Short Form-36

**Figure 2:**
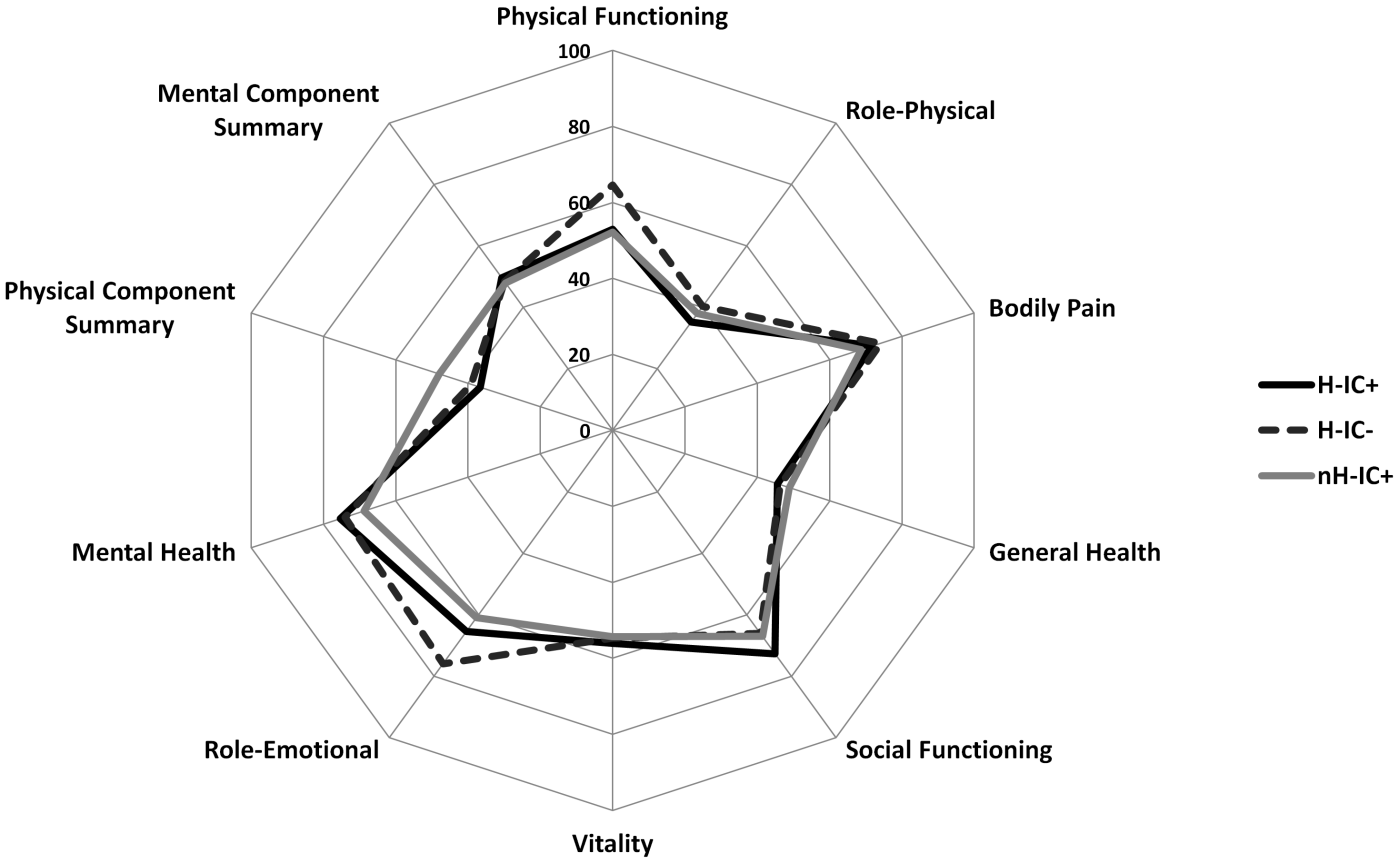
Spider graph of the Short Form-36 measurements median 16 months after ICU discharge, adjusted for covariates. Enclosed as file ‘figure 2’.

H-IC+ respondents reported a similar HR-QoL to H-IC− in almost all domains of the SF-36, with the exception of a difference in the Role Physical dimension namely, H-IC+ reported more problems with work or other daily activities as a result of physical health (*P* = 0.04) (Table 2). Compared to nH-IC+, H-IC+ reported a lower score on the resulting aggregated PCS (*P<0.001*), while the aggregated MCS was not different between the groups. The results for the other domains of the SF-36 were similar (Figure 2). No correlations were found between the PCS and MCS as Pearson correlation coefficients were r = 0.22 for H-IC+ (*P* = 0.30), r = 0.14 for H-IC− (*P* = 0.90) and r = 0.10 for nH-IC+ (*P* = 0.28). Both H-IC+ and H-IC− survivors evaluated their HR-QoL on several domains of the SF-36 worse compared with the age-adjusted general Dutch population of which the reference values are reflected in Table 2.

**Table 2:** Results of Short Form-36 measurements median 16 months after ICU discharge adjusted for covariates.

SF-36†	Haematology patients with ICU admission (N = 27)	Haematology patients without ICU admission (N = 93)	General ICU patients (N = 149)	General Population Subgroup Age 41–60 [11] (reference values)
Physical Functioning	52.9±27.6	64.6±27.6	52.2±32.0	84.0±19.6
Role-Physical	35.2±35.5^a^	40.3±42.8	38.0±41.1	74.5±36.8
Bodily Pain	71.8±25.1	74.1±24.5	68.7±28.5	71.8±24.1
General Health	45.6±21.5	46.3±23.4	48.8±22.0	69.7±20.6
Social Functioning	72.7±26.4	66.1±26.3	67.0±25.8	83.5±22.1
Vitality	56.0±16.7	54.7±20.5	54.3±19.6	68.6±20.2
Role-Emotional	65.4±40.8	76.0±37.9	61.0±43.2	81.6±33.2
Mental Health	75.4±19.0	74.0±17.7	68.8±19.2	75.6±18.5
Physical Component Summary	36.7±8.5	39.1±11.6	48.0±9.7^b^	n.a.
Mental Component Summary	49.6±11.2	48.4±10.7	47.9±9.8	n.a.

Data are expressed as mean with standard deviation (±).

† *adjusted for diagnose acute leukaemia and MDS and LOS-in hospital using log transformed data (not shown)*.

a statistically significantly different (*P*<0.05) compared with haematology patients without ICU admission.

b statistically significantly different (*P*<0.05) compared with haematology patients with ICU admission.

### Checklist individual strength-fatigue

No significant differences between H-IC+ and H-IC− or H-IC+ and nH-IC+ were found for self evaluated fatigue score after adjusting for covariates (Table 3).

**Table 3:** Results of Checklist Individual Strength, Cognitive Failure Questionnaire and Hospital Anxiety and Depression Scale measurements median 16 months after ICU discharge adjusted for covariates.

	Haematology patients with ICU admission (N = 27)	Haematology patients without ICU admission (N = 93)	General ICU patients (N = 149)
**CIS** † ***^-^*** 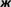			
CIS-total	31.4±12.8	28.8±13.4	33.3±13.9
**CFQ** † ***^-^*** 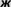			
Memory	7.3±4.1	7.1±4.6	8.2±5.4
Distractibility	10.2±5.9	10.8±5.6	11.7±6.9
Social blunders	6.0±4.1	6.5±3.8	7.9±5.4
Names	3.6±2.0	3.1±2.0	3.2±2.1
CFQ-total	27.5±16.0	26.7±13.8	28.8±16.0
**HADS** † ***^−^*** 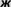			n.a.
Anxiety	5.4±5.2	4.9±4.2	
Depression	4.3±4.1	4.6±3.7	
HADS-total	9.4±8.9	9.5±7.2	

Data are expressed as mean with standard deviation (±)

† *adjusted for diagnose acute leukaemia and MDS and LOS-in hospital using log transformed data (not shown)*.

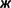
*adjusted for PCS*.

### Cognitive Failure Questionnaire

Cognitive failure was similar after adjusting for covariates on all measured cognitive dimensions between H-IC+ and H-IC−. The overall cognitive functioning tended to be better for H-IC+ than for nH-IC+ (*P* = 0.06) (Table 3).

### Hospital anxiety and depression scale

Adjusted results showed no differences in anxiety or depression between H-IC+ and H-IC− a median 16 months after admission (Table 3). For nH-IC+ were no data available.

## Discussion

The main finding of the present study is that a median of 18 months after admission to an ICU, patients who had been treated for a haematological disease and were admitted to an ICU experienced only a lower quality in the physical aspects of their life compared with those admitted to the ICU without a haematological disease. Patients treated for a haematological malignancy who had not been admitted to the ICU reported only better role-physical functioning. In addition, the long-term health-related mental quality of life as well as fatigue, cognition, anxiety and depression was similar between the groups. As we did not correct for multiple testing, the small differences between groups appear not to be of clinical relevance.

It is likely that complications following haematological treatment and HSCT both influence physical functioning and the ability to perform social activities [17].The fact that the only differences in HRQoL concerned physical aspects may have been due to complications during haematological treatment that necessitated the ICU admission [18].

The recent publication by Oeyen et al [3], in which long-term quality of life was prospectively assessed in haematological patients 1 year after ICU discharge, reports poor QoL outcomes at 1 year, particularly for the haematological subgroup. However, physical complications of haematological treatment are likely to be related to the severity of the underlying disease, its treatment and need for more rigorous ICU therapy and seem to require longer rehabilitation periods than 1 year and also exceeds the median 18 months reported in our current study. This is what may distinguish patients with haematological malignancies from general ICU patients and seems confirmed in a recent study [19] where the PCS at 5 years post-HSCT was similar to those of nH-IC+ in our study at 18 months. These studies represent the limited amount of evidence available on long-term HR-QoL. ICU survival and subsequent hospital survival have improved the past decade to such extent that long-term HR-QoL becomes more important in the decision to admit or not admit a critically ill patient to the ICU. Our finding that long-term HRQoL is comparable in haematological patients with and without an ICU admission is of relevance. This emphasizes the need for more long-term HR-QoL studies that may correct the assumption that patients should not be admitted to the ICU assuming that the long-term outcome will be poor.

The absence of any correlation between the physical and mental component summary scores in all subgroups is remarkable, but has been described earlier [20]. Curbow et al found that recipients of stem cell transplant reported more positive changes in relationship and existential/psychological domains and more negative changes in the physical health domain [21]. The same has been observed for survivors of meningococcal septic shock, who, despite suffering from severe skin scarring or extensive amputation, did not show more behavioural problems as predictor for poorer HRQoL, nor more cognitive dysfunction [22]. In fact, cancer survivorship is a well-recognized phenomenon in which patients confronted with a life threatening illness are faced with the necessity to accommodate to the disease leading to acceptance of and adjustment to the illness [23]. These patients might experience a similar condition higher than they would have if they had not experienced a serious illness [24].

The strength of the current study is the explorative character and clinical relevance of the results, comparing H-IC+ with both H-IC− and nH-IC+ patients. However, there are also relevant limitations that need to be addressed. Despite the high response rate on the questionnaires, the possibility of non-response bias [25] cannot ruled out. Also the study was undertaken in such a specific population that only a relatively small number of patients could be analyzed. As a consequence, it was not feasible to analyze data for the subgroup of allogeneic HSCT recipients who may be more likely to experience treatment-related physical complications such as graft-versus-host-disease (GvHD), that are known to influence HRQoL [26]. The type of conditioning regimen, female gender, younger age, inadequate social support and pre-transplant psychological distress have also been reported to be predictors for a poorer HRQoL following HSCT [27]. Disease status and performance status preceding intensive treatment for haematological diseases can usually be assumed to be good to be qualified, but further baseline characteristics such as social status and pre-existing distress could not be considered as the study was retrospective. Moreover, we only measured HRQoL once where serial measures would have been more informative on changes over time. Nevertheless, patients with haematological malignancies form a unique subpopulation of ICU patients and our cohort represents one of the largest with long-term HRQoL evaluations currently available. This is increasingly becoming considered to represent a relevant measure of outcome.

## Conclusions

In conclusion, there is no support for the assumption that patients undergoing treatment for a haematological malignancy who are admitted to the ICU have a worse long-term HRQoL than those who are not. Therefore the risk of a poorer long term quality of life should no longer be used as an argument not to admit these patients to the ICU.
